# Lateral extra‐articular tenodesis improves functional movement outcomes in ACL reconstruction patients

**DOI:** 10.1002/jeo2.70269

**Published:** 2025-05-19

**Authors:** Luyckx Thomas, Wen Zhishen, Kluyskens Louis, Tampere Thomas, Vanrenterghem Jos, Claeys Kurt

**Affiliations:** ^1^ Department of Orthopedic Surgery AZ Delta Roeselare Roeselare Belgium; ^2^ Department of Rehabilitation Sciences KU Leuven Brugge Belgium; ^3^ Department of Orthopedics Surgery AZ Monica Antwerpen Antwerpen Belgium; ^4^ Department of Orthopaedic Surgery UZ Gent Gent Belgium; ^5^ Department of Rehabilitation Sciences KU Leuven Leuven Belgium

**Keywords:** anterior cruciate ligament, lateral extra‐articular tenodesis, meniscus injury, functional movement outcomes

## Abstract

**Purpose:**

The primary aim was to evaluate the functional impact of adding lateral extra‐articular tenodesis (LET) to an anterior cruciate ligament (ACL) reconstruction (ACLR). The secondary aim was to assess whether LET provides benefits in muscle strength, patient‐reported outcome measures (PROMs) and return to sport (RTS) pass rate, as well as to examine the influence of meniscal injury on performance in ACLR patients.

**Methods:**

A retrospective cohort of prospectively collected data on all ACL procedures was performed at AZ Delta Roeselare between 2017 and 2019. The patients followed a 7‐month rehabilitation programme and were assessed according to standardised RTS criteria. Patients were divided into two groups according to whether they received LET technique or not, and then each group was further divided based on the presence or absence of meniscal injury. The primary outcomes were the functional movement tests including agility t‐test and hop tests. The secondary outcomes were the muscle strength, PROMs and RTS pass rate. A two‐way ANCOVA test or a non‐parametric test was used to investigate clinical and functional differences among the four groups.

**Results:**

A total of 105 ACL injury patients who underwent ACLR with or without LET were included. The cohort consisted of 74 males and 31 females, with an average age of 25.0 ± 6.2 years. Significantly statistical differences among groups were found for the functional movement tests at 7 months post‐operatively in favour of the ACLR with LET groups compared with isolated ACLR groups (agility t‐test: 95% confidence interval [CI] 0.3–1.2, *p* = 0.003; cross‐over hop test: injured leg: 95%CI 20–86, *p* = 0.002; uninjured leg: 95%CI 12–76, *p* = 0.008; triple hop test: injured leg: 95%CI 7–77, *p* = 0.019). There were no significant differences in muscle strength, PROMs or RTS pass rates among the groups. Meniscal injury had no significant effect on any variable of interest.

**Conclusion:**

The addition of a LET procedure to an ACLR showed important improvements in functional movement outcomes at 7 months post‐operatively with no negative effect on muscle strength or PROMs.

**Level of evidence:**

Level III.

**Abbreviations**
ACL: anterior cruciate ligament
ACLR: anterior cruciate ligament reconstruction
iACLR: isolated anterior cruciate ligament reconstruction
LET: lateral extra‐articular tenodesis
LSI: limb symmetry index
PROMs: patient‐reported outcome measures
RTS: return to sport

## INTRODUCTION

Research has shown that residual rotatory knee instability after anterior cruciate ligament (ACL) reconstruction (ACLR) can lead to graft failure [10], osteochondral damage [51] and osteoarthritis of the knee joint [51]. These complications significantly affect patients' daily activity [4] and quality of life [3, 20]. Therefore, it is considered important to minimise residual rotatory knee instability when treating patients with ACL deficiency.

Currently, there is great interest in the role of anterolateral structures of the knee in controlling rotatory knee instability [47, 53]. There is an estimated 11%–79% of acute ACL ruptures that occur with concomitant anterolateral complex injury observed with magnetic resonance imaging [1, 44]. Orthopedic surgeons are therefore increasingly performing a lateral extra‐articular tenodesis (LET) concomitant to ACLR to control the rotatory knee instability [5, 19, 25]. Current in vitro LET technique studies reported that the tenodesis tissue could decrease the stress on the ACL graft by 43% and restore rotatory knee stability, then conferring a protective effect on the knee joint [16, 38, 54]. Recent research by Getgood et al. demonstrated that performing LET on top of ACLR in young patients who were at high risk of failure significantly reduced the frequency of graft rupture and persistent rotatory knee instability at 2 years post‐operatively [23]. Guzzini et al. also noted that the combination of a LET with ACLR in elite female football players demonstrated excellent results in terms of subjective scales and rates of successful return to sport (RTS) [26].

Assessing functional movement after ACLR is crucial for optimising recovery and ensuring a successful RTS. However, despite its potential benefits, functional movement testing is rarely used as a primary outcome measure in ACLR research. Instead, much of the focus has been on structural injuries, such as concomitant meniscal damage, which affects over 50% of ACL injury cases and can accelerate knee degeneration [27]. Additionally, RTS outcomes remain suboptimal, with only 55% of patients returning to competitive sports like football, handball, alpine skiing, and basketball [2]. Given these challenges, incorporating functional movement assessments into post‐ACLR evaluations may provide valuable insights into rehabilitation progress and readiness for RTS.

Therefore, the primary aim was to evaluate the functional impact of adding LET to an ACLR. It was hypothesised that incorporating LET would improve functional outcomes in patients with ACLR. The secondary aim was to assess whether LET provides benefits in muscle strength, patient‐reported outcome measures (PROMs) and RTS pass rate, as well as to examine the influence of meniscal injury on performance in ACLR patients. It was hypothesised that LET would contribute to better muscle strength, PROMs, and RTS success rates, while meniscal injury would negatively affect recovery and performance outcomes.

## MATERIALS AND METHODS

### Patients

This study was reported using the Strengthening the Reporting of Observational Studies in Epidemiology (STROBE) guidelines for cohort studies [58]. The study was approved by our institutional review board and complied with the Declaration of Helsinki. All patients provided informed consent for the use of their data for research and publishing purpose. No external funding was received for the initiation or completion of this study. A retrospective cohort analysis was performed using the data of ACLR patients between 2017 and 2019 and prospectively followed up for 7 months.

Patients were included in the study if they (1) had an ACL‐deficient knee requiring surgery; (2) were no more than 40 years old at the time of surgery and (3) underwent ACLR using hamstring tendon autograft. Patients were excluded if they (1) had undergone previous ACLR in either knee; (2) underwent concomitant procedures involving the posterior cruciate ligament or the medial/lateral collateral ligament of the knee; (3) had cartilage injury greater than grade 2 using the Outerbridge grading system; [52] (4) had knee osteoarthritis greater than Grade 1 using the Kellgren–Lawrence grading system [34].

Patients were allocated to receive the LET technique based on clinical decision‐making criteria. Factors such as age, ligamentous laxity and activity level were considered to determine the necessity of additional stabilisation provided by LET. A high risk of reinjury was defined as the presence of two or more of the following criteria: (1) young age ( < 25 years old), (2) high grade pivot shift, (3) involvement in pivoting sports and (4) general hyperlaxity. All patients were distributed into four groups: (1) patients who underwent ACLR combined with LET and meniscal surgery; (2) patients who underwent ACLR combined with LET; (3) patients who underwent isolated ACLR (iACLR) combined with meniscal surgery; and (4) patients who underwent iACLR.

### Surgical techniques and rehabilitation programme

All the surgical procedures were performed by experienced orthopaedics (TL and AR). Each patient underwent anatomical single‐bundle ACLR, utilising hamstring tendon autograft. Firstly, the tendons were harvested through a small paramedial tibial incision. The femoral and tibial tunnel were then positioned in the original ACL footprint. Finally, the graft was fixed on the femur with a tightrope (Arthrex, Inc. Naples, Florida) and with an interference screw (RCI; Smith and Nephew, USA) and fixation post or staple [8].

For the patients performed with additional LET technique, the Cocker‐Arnold or the mini‐monoloop technique was performed [18, 40]. Both techniques were used depending on the surgeon's preference. Firstly, one 6‐cm longitudinal incision was made on the lateral knee joint. When using the mini‐monoloop technique, a 1.0 × 12 cm strip of iliotibial band was created. Next, the iliotibial band graft was passed beneath the fibular collateral ligament. Finally, the iliotibial band was fixed in the over the top position according to Krackov in a bone tunnel with interference screw [40].

In the Cocker‐Arnold procedure, an 8‐cm‐long by 1‐cm‐wide strip of iliotibial band was harvested. Then, the iliotibial tract strip was twisted 180° on top of the FCL and sutured under tension with periosteal stitches to Gerdy's tubercle insertion [57].

Meniscal tears were also diagnosed during arthroscopy under direct visualisation. For patients with meniscal lesions, the meniscus was repaired or reshaped (meniscoplasty), or part of the meniscus was removed (partial meniscectomy) according to the injury status.

A standardised post‐operative physical therapy protocol was followed over 7 months for all the patients with a self‐chosen physiotherapist. Full weight bearing and restoration of range of motion were important goals in the early 4–6 weeks after surgery. Gradual RTS activities were allowed starting at 3 months for non‐pivoting sports involving jogging and running, and at 5 months for pivoting sports involving jumping and agility test. RTS or full activity was allowed after achieving appropriate clinical and functional standards in muscle strength training, functional movement tests and PROMs [12, 13].

### Clinical and functional assessment

To evaluate knee function and subjective instability, functional movement tests such as the agility t‐test, cross‐over hop test and triple hop test were performed as part of the standardised rehabilitation protocol [13, 21, 49]. The agility‐T test is a test which evaluates agility during running with changes of direction. Four cones are set up in a T‐shape. The subject has to sprint and shuffle sideways as fast as possible from cone to cone, then back to the start cone. The stopwatch is stopped when the subject passes the first cone again. The patient is allowed to perform the test three times, the fastest attempt counts. The cut‐off score is 11.5 s for male subjects and 12.5 s for female subjects. When the patient is slower, the score is considered as poor [50]. Triple and cross‐over hop tests for distance were used. These tests measure the distance that an individual can cover while jumping on one leg. The patient performs three consecutive jumps as far as possible using the same foot. For cross‐over hop each patient does 3 hops while crossing a line drawn on the floor. Each patient is allowed three attempts with both the right and left foot. Then the limb symmetry index (LSI) was calculated from the mean results of the affected and non‐affected side. Equal to or more than 90% of LSI in the hop tests is recommended for returning to sport [7, 35].

For the Biodex strength tests, an isokinetic dynamometer was used. The concentric strength was measured for the quadriceps and hamstrings. The concentric tests of the quadriceps and hamstrings were performed at speeds of 60°/s and 240°/s. RTS criteria require equal to or more than 90% of LSI in muscle strength at a speed of 60°/s [7, 35].

Subjective outcomes were assessed using the knee injury and osteoarthritis outcome score, and the anterior cruciate ligament‐return to sport after injury questionnaire. The RTS criterion was considered to achieve more than a 90% scores on the knee injury and osteoarthritis outcome score [35].

The PROMs, muscle strength and functional movement tests were measured at 3‐month, 5‐month and 7‐month post‐operatively, potentially leaving out some of the tests depending on what the patient was able to do at that time. The patients passing RTS criteria could RTS or if failing needed to continue their rehabilitation programme until passing.

### Statistical analysis

For data analysis, patients were divided into 4 groups based on the surgical procedure and meniscal injury: ACLR with LET with meniscal injury, ACLR with LET without meniscal injury, iACLR with meniscal injury and iACLR without meniscal injury. For some missing data (no more than 10% of the total) in each category (functional tests, PROMs, and muscle strength), the database was imputed using the multiple imputation method. Descriptive statistics were calculated for demographic and clinical factors. For qualitative data, this included the number and percentage of patients. For quantitative data, this included the mean and standard deviation. The normality of variables was assessed with the Shapiro–Wilk test. Chi‐quare test was used to assess for differences in qualitative variables among the four groups. For quantitative variables, a two‐way ANCOVA test or Kruskal–Wallis test was used to investigate differences among the groups. In the two‐way ANCOVA test, type of surgery and meniscal injury were the two main effect factors, and the gender was a covariate. Those two main effects and one interaction effect were tested to investigate differences among the groups. Statistical significance was set at *p* < 0.05. All statistical analyses were performed using SPSS version 26 (IBM, New York, NY). A post hoc power analysis was performed using G*Power 3.1 [17]. Consequently, it was shown that the sample size of 105 in this study could achieve a power of 0.82 with an *α* of 0.05 for the functional outcomes.

## RESULTS

**Figure 1:**
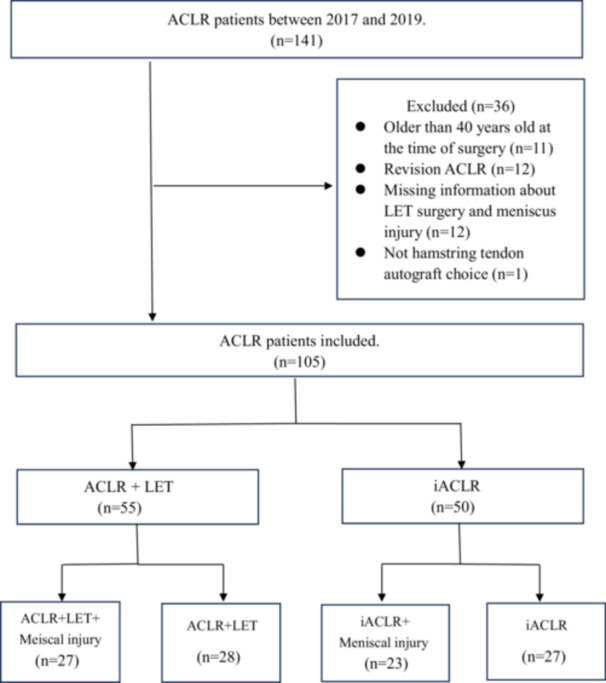
Flow chart of the numbers of ACLR patients that were excluded and included. ACLR, anterior cruciate ligament reconstruction; iACLR, isolated anterior cruciate ligament; LET, lateral extra‐articular tenodesis.

A total of 105 subjects were eligible for inclusion in this study during a 7‐month follow‐up between 2017 and 2019 (Figure 1). The demographic data for patients among the four groups included in analysis are shown in Table 1. There were significant gender differences among the four groups, with the proportion of males in the additional LET groups being significantly higher than that in the iACLR group (*p* = 0.001). No statistically significant difference was found in age, weight or activity at time before injury [30] among these four groups. For patients with meniscal injury, the majority underwent meniscectomy in the ACLR with LET group and iACLR group.

**Table 1:** Demographics data for ACLR subjects.

	ACLR + LET		iACLR		*p*‐value
	With meniscal injury	Without meniscal injury	With meniscal injury	Without meniscal injury	
Number of subjects (*n*)	27	28	23	27	–
Age (years, mean ± SD)	23.5 ± 3.8	24.6 ± 5.3	26.5 ± 7.7	25.9 ± 7.4	0.427*
Sex (%, male)	24 (89%)	24 (86%)	13 (57%)	13 (48%)	**0.001** **
Weight (kg)	74.8 ± 10.0	78.6 ± 11.3	75.2 ± 16.1	72.1 ± 13.4	0.180*
Activity at time before injury (*n*)					0.119**
Pivoting sport	22	25	16	20	
Non‐pivoting sport	1	1	4	0	
Other physical activity	4	2	3	7	
Location of meniscus tear (*n*)					–
Medial	8	–	11	–	
Lateral	15	–	10	–	
Medial + Lateral	4	–	2	–	
Meniscal procedure (*n*)					–
Meniscectomy	17	–	15	–	
Meniscoplasty	5	–	5	–	
Meniscectomy + Meniscoplasty	3	–	2	–	

*Note*: (Pivoting sport: American football/rugby, basketball, dancing, floorball, gymnastics, handball, ice hockey/bandy, martial arts, racket sports, football, volleyball, wrestling. Non‐pivoting sport: cross‐country skiing, cycling, horseback riding, motocross/enduro, skateboarding, snowboarding and surfing/wakeboarding. Other physical activity: other recreational sport, exercise, trampoline). Meniscoplasty: This surgery aims to repair or reshape the damaged meniscus, preserving as much of the meniscus as possible.

Abbreviations: ACLR, anterior cruciate ligament reconstruction; iACLR, isolated anterior cruciate ligament reconstruction; LET, lateral extra‐articular tenodesis; SD, standard deviation.

* Non‐parametric statistical comparison.

** Chi‐square statistical comparison.

In the two‐way ANCOVA test, the effect of bias was reduced by controlling for gender as a covariate. Procedure type was a significant main effect for functional movement outcomes. Patients who underwent ACLR with LET performed the agility T‐test significantly faster than those in the iACLR groups (*p* = 0.003) (Table 2). Additionally, ACLR with LET patients demonstrated superior performance in the cross‐over hop and triple hop tests compared to iACLR patients, with statistically significant differences observed for both the injured and uninjured legs (*p*‐values ranging from 0.002 to 0.019) (Table 2). Interestingly, there were no group differences in LSI for the two hop tests. Meniscal injury was not a significant main effect for any variable and there was no interaction effect between procedure type and meniscal injury for any of the three functional outcome tests.

**Table 2:** Summary of the functional movement tests at 7 months post‐op.

Test	Leg	ACLR + LET		iACLR		Interaction effect	Procedure type effect	Meniscal injury effect
		With meniscal injury	Without meniscal injury	With meniscal injury	Without meniscal injury			
Agility t‐test (s)*		**11.1** ± **1.1**	**11.5** ± **1.2**	**12.7** ± **1.2**	**12.3** ± **1.6**	*F* = 3.81, *p* = 0.054	***F*** = **9.10, *p*** = **0.003**	*F* = 0.18, *p* = 0.671
Triple hop for distance (cm)*								
	Injured	**488** ± **97**	**447** ± **75**	**386** ± **91**	**404** ± **99**	*F* = 3.69, *p* = 0.057	***F*** = **5.68, *p*** = **0.019**	*F* = 0.17, *p* = 0.682
	Uninjured	491 ± 105	459 ± 75	414 ± 80	411 ± 87	*F* = 1.24, *p* = 0.268	*F* = 3.15, *p* = 0.079	*F* = 0.64, *p* = 0.425
	LSI*	100 ± 8%	98 ± 8%	93 ± 12%	98 ± 10%	*F* = 3.68, *p* = 0.058	*F* = 3.43, *p* = 0.067	*F* = 0.26, *p* = 0.612
Cross‐over hop for distance (cm)*								
	Injured	**459** ± **78**	**440** ± **82**	**361** ± **74**	**380** ± **100**	*F* = 1.84, *p* = 0.178	***F*** = **10.17 *p*** = **0.002**	*F* = 0.07, *p* = 0.788
	Uninjured	**475** ± **75**	**448** ± **82**	**384** ± **71**	**391** ± **102**	*F* = 1.68, *p* = 0.199	***F*** = **7.43, *p*** = **0.008**	*F* = 0.10, *p* = 0.757
	LSI*	97 ± 6%	98 ± 8%	94 ± 8%	97 ± 8%	*F* = 0.21, *p* = 0.650	*F* = 2.89, *p* = 0.092	*F* = 2.62, *p* = 0.109

*Note*: Statistically significant differences in the two‐way ANCOVA statistical analysis are denoted by bold characters.

Abbreviations: ACLR, anterior cruciate ligament reconstruction; ANCOVA, analysis of covariance; cm, centimetre; iACLR, isolated anterior cruciate ligament reconstruction; LET, lateral extra‐articular tenodesis; LSI, limb symmetry index; s, second.

* Two‐way ANCOVA statistical comparison.

There were no significant differences among the groups in PROMs and muscle strength (Table 3, Table 4). There were also no differences among the groups in the number of patients who had passed RTS criteria as classified by their scores of functional tests, muscle strength, and PROMs (Table 5).

**Table 3:** Summary of patient reported outcome measures at 7 months post‐op.

Questionnaire	ACLR + LET		iACLR		Test statistics	*p*‐value
	With meniscal injury	Without meniscal injury	With meniscal injury	Without meniscal injury		
ACL‐RSI (%)	72 ± 21	71 ± 20	62 ± 18	72 ± 18	5.34	0.149
KOOS subscale scores						
Pain (%)	91 ± 11	92 ± 6	93 ± 7	90 ± 11	1.74	0.628
Symptoms (%)	85 ± 14	82 ± 16	86 ± 9	85 ± 7	1.74	0.628
Daily life (%)	97 ± 7	97 ± 3	96 ± 4	97 ± 4	5.76	0.124
Sport (%)	85 ± 15	84 ± 14	79 ± 14	86 ± 10	5.09	0.166
Quality of life (%)	82 ± 20	78 ± 15	77 ± 18	80 ± 17	3.74	0.290

*Note*: No significant differences were observed with non‐parametric statistical analysis.

Abbreviations: ACLR, anterior cruciate ligament reconstruction; ACL‐RSI, anterior cruciate ligament‐return to sport after injury; iACLR, isolated anterior cruciate ligament reconstruction; KOOS, Knee injury and Osteoarthritis Outcomes Score; LET, lateral extra‐articular tenodesis.

**Table 4:** Limb symmetry index of the knee hamstring and quadriceps at two angular velocities at 7 months post‐op.

Angular · Velocity (°/s)	LSI	ACLR + LET		iACLR		Interaction effect	Procedure type effect	Meniscal injury effect
		With meniscal injury	Without meniscal injury	With meniscal injury	Without meniscal injury			
60								
	H	84 ± 10%	89 ± 14%	86 ± 12%	85 ± 11%	*F* = 1.94, *p* = 0.167	*F* = 0.03, *p* = 0.872	*F* = 0.41, *p* = 0.522
	Q	84 ± 13%	82 ± 14%	81 ± 14%	83 ± 12%	*F* = 0.61, *p* = 0.437	*F* < 0.01, *p* = 0.974	*F* = 0.01, *p* = 0.926
240								
	H	89 ± 10%	95 ± 18%	93 ± 14%	93 ± 14%	*F* = 0.73, *p* = 0.396	*F* = 0.10, *p* = 0.754	*F* = 1.10, *p* = 0.296
	Q	89 ± 11%	89 ± 12%	84 ± 21%	88 ± 11%	*F* = 0.74, *p* = 0.390	*F* = 0.44, *p* = 0.511	*F* = 0.72, *p* = 0.400

*Note*: No significant differences were observed with two‐way ANCOVA statistical analysis.

Abbreviations: ACLR, anterior cruciate ligament reconstruction; H, hamstring; iACLR, isolated anterior cruciate ligament reconstruction; LET, lateral extra‐articular tenodesis; LSI, limb symmetry index; Q, quadriceps.

**Table 5:** The number of patients passing RTS criteria at each group and results of chi‐square comparison.

Categorisation	ACLR + LET		iACLR		*p*‐value
	With meniscal injury (*n* = 27)	Without meniscal injury (*n* = 28)	With meniscal injury (*n* = 23)	Without meniscal injury (*n* = 27)	
Functional movement test	14 (52%)	15(54%)	5 (22%)	12 (44%)	0.096
Muscle strength	2 (7%)	3 (11%)	3 (13%)	5 (19%)	0.652
PROMs	25 (93%)	28 (100%)	20(87%)	24 (89%)	0.291

*Note*: No significant differences were observed with Chi‐square statistical analysis.

Abbreviations: ACLR, anterior cruciate ligament reconstruction; iACLR, isolated anterior cruciate ligament reconstruction; LET, lateral extra‐articular tenodesis; PROMs, patient reported outcome measures; RTS, return to sport.

## DISCUSSION

The most important findings of this study were that patients who underwent ACLR + LET had significantly improved functional movement outcomes at 7 months post‐operatively compared to patients only receiving iACLR, and that meniscal injury had no negative impact on performance in patients undergoing ACLR. The finding was somewhat surprising as the addition of an extra procedure during surgery also poses some risks and might make rehab more challenging for these patients. Moreover, the LET procedure was performed in the high‐risk patients and the high‐grade pivot shifts that are more prone to residual rotatory knee instability. Despite this, the functional performance of the LET patients was better than their non‐LET counterparts. We hypothesised that the outcomes of functional movement testing would be better in ACLR + LET groups compared to iACLR groups, and this primary hypothesis was supported by our findings. We disproved our secondary hypothesis that LET would help improve muscle strength, PROMs, and RTS success rates, whereas meniscal injury would impair performance in a rehabilitation programme in patients with ACL injury.

The results suggest a greater dynamic knee joint stability can be achieved with the addition of the LET. Returning to competitive sports requires good knee stability, several studies have shown that LET surgery could improve dynamic knee rotatory stability [23, 26, 42, 43]. Perelli et.al also reported that additional LET can improve kinematic outcomes of single‐leg hop test and quantitative pivot‐shift test at a 2‐year follow‐up [43]. Ferretti et al. even reported a graft failure rate of 11.4% in patients undergoing iACLR but none in those undergoing combined ACLR + LET at a minimum follow‐up of 10 years [18]. A study on the reliability and validity of hop tests after ACLR reported standard errors of measurements and minimal detectable changes for LSI values [45]. These values suggest that changes within this range may be necessary to be confident that a real change in performance has occurred, though they do not directly equate to minimal clinically importance difference. However, specific thresholds for the minimal clinically importance difference in hop tests and agility T‐test for ACL surgery patients are not well‐established in the current literature.

In this study, there were no significant differences among the four groups in muscle strength, PROMs and RTS pass rate. Returning to sport requires adequate lower extremity muscle strength and good PROMs scores for ACL injury patients to minimise the risk of re‐injury [14, 55]. Some longer follow‐up studies demonstrated similar PROMs between additional LET groups and iACLR groups [28, 32, 37]. Moreover, Getgood et.al reported that quadriceps muscle strength remained the same in both iACLR group and the additional LET group at 12 months post‐operatively [22]. However, the association between LET technique and muscle strength recovery is unclear, as muscle strength is most affected by graft choice [11, 31, 36]. It was reported that PROMs and RTS rates did not differ between graft options, but the postoperative extensor strength was reduced in patients with bone‐patella‐tendon‐bone and quadriceps tendon autograft whereas flexion strength was weakened in patients with hamstring tendon [41, 48]. Regarding the RTS pass rate, Rezansoff et.al also reported similar results in the ACLR with LET group compared with the iACLR group in young, active patients who played high‐risk sports [46].

The results of this study showed that meniscal injury had no negative effect on performance outcomes in ACLR patients. This is in contrast to some studies that muscular strength of limbs with meniscal injuries was significantly weaker [15]. However, Biswal et. al reported that associated meniscal injury does not affect functional outcome in ACLR patients [6]. Several studies have also reported that meniscal treatment did not show significant improvement in persistent weakness, asymmetry or muscle recovery for ACL injury patients or patients with combined ACL and meniscal injury [9, 24]. Furthermore, in this study, most patients underwent meniscectomy rather than repair, which may contribute to the findings, as meniscal repair does slow the immediate postoperative rehabilitation, thereby delaying full recovery and even muscle strength [39].

Overall, this study shows that the addition of LET technique in ACLR surgery has a positive influence on the functional movement outcomes at 7 months post‐operatively. In contrast, it also demonstrates that most patients did not meet several RTS criteria which suggests that most of the ACLR patients were not ready yet to resume high impact or pivoting sports activities at this point. In our view, longer rehabilitation and later RTS is indicated [13]. It also provides new ideas into how LET technology can improve movement quality through kinematics and help patients better return to pre‐injury or competitive sports level [56].

The present study certainly has some limitations. Firstly, the study was retrospective in nature, so the analysis was limited to the data available in the medical record database. Secondly, this study lacks patients' height data, which may have a potential impact on certain biomechanical assessments. However, given that the primary evaluation indicators in this study (functional movement testing) are predominantly influenced by factors such as surgical procedures and rehabilitation training, the absence of height data is unlikely to have a significant effect on the main research conclusions. The third is that heterogeneity among meniscal pathology subgroups is not discussed, limiting the ability to exclude meniscal injury effects. Fourthly, the indication for additional LET technique in this study was based on surgical judgement and was therefore subject of selection bias as the LET was only performed in the high‐risk patients and the high‐grade pivot shifts. Today, there is still no consensus on objective measures when to use the LET technique. Currently, positive pivot‐shift test ( ≥ Grade 2) is the most common reason orthopedics chose to add LET to ACLR, with revision ACLR, patient age younger than 25 years, and general sports participation following closely behind [29]. The best indication to perform a LET procedure still needs to be investigated. However, despite this potential selection bias, the LET group outperformed the iACLR group. Fifthly, the functional movement tests in this study evaluate anticipated movements but in real sports unanticipated movements are more common and considered more challenging. It is more consistent with reality if some unanticipated movement tests are also included. Sixthly, the short‐term follow‐up nature of this study limits further research on the advantages and disadvantages of additional LET procedures. In the long term, it remains to be seen whether patients who do not undergo LET will perform as well as those who do. Finally, Tegner scores [33] were not available in this study and therefore the activity level of the groups could not be compared, which could influence the results. Despite the above limitations, we believe that the increasing popularity of the LET procedure justifies a need for advancing insights into its added value for patient functionality. Our study demonstrated that the LET technique improves functional movement outcomes in patients following ACLR, and it is independent of meniscal injuries.

## CONCLUSION

The main finding of this study was that the addition of a LET in ACLR patients increased their functional outcomes after 7 months with no negative effect on muscle strength and PROMs. Future research is needed to determine whether the indication for adding a LET in patients with ACLR could be broadened.

## AUTHOR CONTRIBUTIONS

**Luyckx Thomas**: Data acquisition; data analysis; data interpretation; drafting manuscript. **Wen Zhishen**: Design; data analysis; data interpretation; drafting manuscript. **Kluyskens Louis**: Data acquisition. **Tampere Thomas**: Data acquisition. **Vanrenterghem Jos**: Manuscript revision. **Claeys Kurt**: Design; data interpretation; manuscript revision.

## CONFLICT OF INTEREST STATEMENT

The authors declare no conflict of interest.

## ETHICS STATEMENT

The institutional review board approved the study, according the Helsinki guidelines (IRB reference number: B1172020000026). Informed consent was obtained from each patient for study participation.

## Data Availability

The data sets generated during the current study are available from the corresponding author on reasonable request.
